# Multiple-Kernel Support Vector Machine for Predicting Internet Gaming Disorder Using Multimodal Fusion of PET, EEG, and Clinical Features

**DOI:** 10.3389/fnins.2022.856510

**Published:** 2022-06-30

**Authors:** Boram Jeong, Jiyoon Lee, Heejung Kim, Seungyeon Gwak, Yu Kyeong Kim, So Young Yoo, Donghwan Lee, Jung-Seok Choi

**Affiliations:** ^1^Department of Statistics, Ewha Womans University, Seoul, South Korea; ^2^Department of Psychiatry, Samsung Medical Center, Seoul, South Korea; ^3^Department of Nuclear Medicine, SMG-SNU Boramae Medical Center, Seoul, South Korea; ^4^Institute of Radiation Medicine, Medical Research Center, Seoul National University, Seoul, South Korea; ^5^Department of Psychiatry, SMG-SNU Boramae Medical Center, Seoul, South Korea

**Keywords:** internet gaming disorder, integrative analysis, multimodal, kernel support vector machine, Positron Emission Tomography, electroencephalography

## Abstract

Internet gaming disorder (IGD) has become an important social and psychiatric issue in recent years. To prevent IGD and provide the appropriate intervention, an accurate prediction method for identifying IGD is necessary. In this study, we investigated machine learning methods of multimodal neuroimaging data including Positron Emission Tomography (PET), Electroencephalography (EEG), and clinical features to enhance prediction accuracy. Unlike the conventional methods which usually concatenate all features into one feature vector, we adopted a multiple-kernel support vector machine (MK-SVM) to classify IGD. We compared the prediction performance of standard machine learning methods such as SVM, random forest, and boosting with the proposed method in patients with IGD (*N* = 28) and healthy controls (*N* = 24). We showed that the prediction accuracy of the optimal MK-SVM using three kinds of modalities was much higher than other conventional machine learning methods, with the highest accuracy being 86.5%, the sensitivity 89.3%, and the specificity 83.3%. Furthermore, we deduced that clinical variables had the highest contribution to the optimal IGD prediction model and that the other two modalities were also indispensable. We found that more efficient integration of multimodal data through kernel combination could contribute to better performance of the prediction model. This study is a novel attempt to integrate each method from different sources and suggests that integrating each method, such as self-administrated reports, PET, and EEG, improves the prediction of IGD.

## Introduction

In modern society, the Internet has become an essential tool for life, and Internet-based games have also become popular for their accessibility and entertainment as a result. On the other hand, various side effects have also increased significantly, and Internet gaming disorder (IGD) has thus become one of the most discussed psychological issues. IGD is caused by repetitive use of internet-based games that leads to significant problems with functioning in the Diagnostic and Statistical Manual of Mental Disorders (DSM-5) which contains preoccupation or obsession, withdrawal symptoms, and overuse (American Psychiatric Association, 2013). According to DSM-5, gaming causes significant impairment or distress in several aspects of a person's life. In addition, the World Health Organization recently recognizes IGD to be a severe public health issue, and IGD has been categorized as a gaming disorder in the International Classification of Diseases 11th Revision (World Health Organization, 2019). Therefore, it is important to properly diagnose and intervene the development of IGD, which requires an accurate prediction method for identifying IGD.

There are several techniques to evaluate and predict IGD. In clinical practice, the most common method for evaluating IGD is self-administrated questionnaires. Patients with IGD usually self-report their Internet gaming usage patterns and the severity of IGD symptoms. For example, clinical and psychological features such as depression, hostility, and life satisfaction are all possible risk factors for IGD (Young and Rogers, 1998; Bargeron and Hormes, 2017). Ko et al. (2007) reported that high exploratory excitability, low reward dependence, low self-esteem, low family function, and online game playing predicted the emergency of Internet addiction. Furthermore, low hostility and low interpersonal sensitivity predicted remission of Internet addiction. Adolescents with IGD showed that attention problems mechanism and social vulnerability mechanism explained the increase in IGD symptoms (Peeters et al., 2018). However, there will be clear limits to understanding or predicting IGD since it relies on subjective responses. In addition, individuals with addictions have poor insights into their problems and underestimate their addictive behaviors.

To overcome the limitations of self-report, neuroimaging methods have become an increasingly important tool for studying brain functions and neuropsychiatric disorders (Volkow et al., 2014). One of the commonly used tools is electroencephalography (EEG). An EEG technique shows the electrical activity of the brain and provides a measure of baseline or underlying neural states before processing information. It has several advantages in that it provides higher temporal resolution images in the brain, less invasiveness for subjects, and higher cost-effectiveness than the other techniques (Waldemar et al., 2007). A previous study showed that absolute powers measured by EEG had discriminating values for patients with IGD and alcohol use disorder (Son et al., 2015). Park et al. (2017) reported that an increase in the fast phasic synchrony of gamma coherence might be a core neurophysiological feature of IGD. The limitation of EEG, however, is that it has a poor spatial resolution, which means that it cannot precisely locate fired neurons in the brain, especially in deeper, older structures (Morin, 2011).

Another neuroimaging method is a Positron Emission Tomography (PET). The PET is used to monitor cerebral blood flow and glucose/oxygen metabolism to provide information on specific molecules such as transporters or receptors and cellular processes including neurotransmitter synthesis and release (Tian et al., 2014). One uniquely valuable PET tool is 18F-fluorodeoxyglucose (18FDG-PET), a radiotracer that measures brain glucose metabolism (Fowler and Ido, 2002). A study with PET reveals that functional changes in a certain cortex could underscore a mechanism that relates to loss of control behavior for IGD subjects (Tian et al., 2014). In the 18F-FDG-PET study, patients with IGD showed hypometabolism in the anterior cingulate cortex (ACC), temporal, frontal, parietal, and striatum, where negative correlations between ACC and game duration and between orbitofrontal cortex and impulsivity occurred (Kim et al., 2019). However, PET is more invasive and time-consuming due to the need for isotope injection (Duc et al., 2020). When compared to EEG, it is not an optimal tool for recording temporal patterns of neuronal activity (Shah et al., 2017). In summary, self-administrated reports, EEG, and PET each have their sources, characteristics, and complementary information.

However, there are few studies on the IGD prediction model based on those findings. Most features associated with IGD found in previous studies are based on group comparisons as mentioned earlier, so findings may include false positives as a result of the multiplicity issue. In addition, the number of features found in each domain's study is very small, and individual studies can be relatively weak signals, making it difficult to construct prediction models purely dependent on these signals. Therefore, the limitation is not solved to which extent those methods provide complementary information that could be introduced to improve the performance when these methods are combined. Researchers began combining multiple techniques, referred to as multimodal neuroimaging to compensate for the limitations of each modality. Multimodal neuroimaging is an approach combining data sets obtained using two or more unimodal modalities, such as MRI and EEG integration, to yield more informative, consistent, and reliable results (Rosa et al., 2010).

There have been several studies aimed at classifying patients with psychiatric disorders using a multimodal neuroimaging approach. Yang et al. (2016) combined connectivity features from resting-state functional Magnetic Resonance Imaging (MRI) and anatomical features of structural MRI data selected by independent component analysis (ICA) in patients with schizophrenia and healthy controls. They showed that a combination of modalities (77.91%) yielded higher accuracy than using a single modality (72.09%). A combination of resting-state functional MRI and magnetoencephalography (MEG) differentiated schizophrenia and healthy controls with an accuracy of 87.91% (Cetin et al., 2016). In the case of depressive disorder, Schmaal et al. (2015) used a combination of functional and structural MRI of different types of patients with major depressive disorder (MDD) and classified chronic and remitted MDD with 62% accuracy, chronic and gradually improved MDD with 61% accuracy, and gradually improved and remitted MDD with 44% accuracy.

Multivariate machine learning approaches can help us predict and classify psychiatric disorders using multimodal neuroimaging. Among the machine learning-based classification methods, the Support Vector Machine (SVM) is commonly used for dealing with multimodality (Tulay et al., 2019). Multiple-kernel SVM enables the contribution of each modality to the classification result to be controlled more closely and potentially improves the power of the SVM algorithm to use complementary information provided by the modalities within its model (Sonnenburg et al., 2006; Dyrba et al., 2012). In the previous study, Dyrba et al. (2015) reported that the integrating multimodal MRI data showed improved classification accuracy compared to utilizing the best single measures by multiple-kernel SVM. In the study with IGD, multiple physiological markers, such as electrooculogram (EOG), photoplethysmogram (PPG), and electroencephalogram (EEG), were utilized to classify individuals who seldom play games, those who enjoy and play games frequently, and those who have IGD (Ha et al., 2021). According to a two-layer feedforward neural network model, the combination of three physiological signals had a higher classification accuracy (90%) than the combination of EOG and PPG or EEG only. Nevertheless, research on the classification of IGD using multimodal neuroimaging approaches is still insufficient.

To the best of our knowledge, however, there have been no studies using multimodal neuroimaging approaches with PET and EEG in addictive disorders. Therefore, in this study, our goal was to find a prediction rule with high prediction accuracy by integrating weak modalities to complement each other and simultaneously take advantage of each unique characteristic to have enough information for prediction in patients with IGD and healthy controls. Using multiple-kernel SVM, we integrated multimodal data consisting of three modalities: PET, EEG, and clinical feature. We further identified the prediction accuracy of multiple-kernel SVM by comparing it with other existing methods including SVM, Xgboost, Random Forest, and deep learning. We hypothesized that multiple-kernel SVM would produce more accurate predictions in the test sample and show less evidence of overfitting compared with other methods.

## Materials and Methods

### Participants

Fifty-two male adults aged 18–34 years were recruited from the SMG-SNU Boramae Medical Center and the surrounding community in Seoul, South Korea. They did not have a history of significant head injury, seizure, or intellectual disability [intelligence quotient (IQ) > 80] (Yeom et al., 1992), or psychotic or neurological disorders and were medication-naïve and right-handed. IGD was diagnosed by trained clinicians based on DSM-5 criteria; participants who spent more than 4 h per day and 30 h per week playing Internet games were included in the IGD group. Young's Internet Addiction Test (Y-IAT) was used to assess the severity of IGD. All HC were recruited from the local community and universities, and none had a history of any psychiatric disorder and all played Internet games for less than 2 h per day. In total, 28 IGD patients and 24 healthy controls (HCs) were included in the present study. Participants visited the SMG-SNU Boramae Medical Center twice in 2 weeks. All subjects received an explanation about the research and were provided written informed consent before participation. They completed EEG, PET, neurocognitive functional test, and a self-administered questionnaire and got monetary reward for participation. The study was conducted following the Declaration of Helsinki. This study was approved by the Institutional Review Board of the SMG-SNU Boramae Medical Center, Seoul and the Republic of Korea.

### Clinical Features

#### Young's Internet Addiction Test

The severity of IGD is assessed by the Young's Internet Addiction Test (Y-IAT) developed by Young (1998). It contained questions including “How often do you find that you stay on-line longer than you intended?” and “How often do you neglect household chores to spend more time on-line?” and were rated on a 5-point scale (from “1 = very rarely” to “5 = very frequently”).

#### Aggression Questionnaires

The Aggression Questionnaires (AQ) consists of 29 questions that assess aggression on a 5-point Likert scale (Buss and Perry, 1992). Participants had to indicate to what extent the statement applied to them (1 = extremely uncharacteristic of me to 5 = extremely characteristic). The instrument provides measures of physical aggression, verbal aggression, and hostile aggression and anger.

#### Behavioral Inhibition System/Behavioral Activation System Scales

The BIS and the BAS scales were utilized to assess sensitivity to punishment Behavioral Inhibition System (BIS) and rewards, respectively Behavioral Activation System Scales (BAS) (Carver and White, 1994). They together consist of 20 items rated on a 4-point scale from “totally agree” to “totally disagree.” The BIS scale contains seven items concerning anticipated punishment.

#### Barratt Impulsiveness Scale-11

The BIS assesses a range of impulsive tendencies using a 4-point scale ranging from 1 (rarely/never) to 4 (almost always/always) (Lee, 1992). Barratt Impulsiveness Scale-11 (BIS-11) has three subscales that assess cognitive impulsivity, motor impulsivity, and non-planning impulsivity (Patton et al., 1995). This instrument has yielded positive correlations with neuropsychological measures of impulsivity and is sensitive to executive function deficits in the prefrontal and orbitofrontal systems in multiple clinical samples (Barratt, 1985; Spinella, 2004).

#### Emotional Control Questionnaire

The Emotional Control Questionnaire (ECQ) evaluates emotional control and aggressive control as a measure of emotional control (Roger and Nesshoever, 1987). It consists of 28 items that answer yes or no. The lower score means the greater the tendency to suppress emotional expression.

#### Beck Depression Inventory

The Beck Depression Inventory (BDI) consists of four statements indicating different levels of the severity of a particular symptom experienced during the past week (Beck et al., 1996). This scale measures the existence and severity of symptoms of depression. A total score of 0–13 is considered minimal depression, 14–19 mild depression, 20–28 moderate depression, and 29–63 severe depression.

#### Beck Anxiety Inventory

The Beck Anxiety Inventory (BAI) uses a 4-point scale (0 = “not at all” to 3 = “severely, it bothered me a lot”) to measure an individual's anxiety (Beck et al., 1988). Scores for the 21 items are summed to yield a single anxiety score. It is a 21-question questionnaire used for measuring how the subject has been feeling in the last week, focusing primarily on somatic symptoms.

#### Psychosocial Wellbeing Index

The stress level was measured with a Psychosocial Wellbeing Index (PWI) which contains 45 items (Kim, 1999). PWI contains questions about physical and psychological status over the last few weeks, covering social role performance, self-confidence, depression, sleep disturbance, anxiety, and the general well-being of respondents. Scores range from 0 to 135 with higher scores indicating higher distress symptoms. Higher scores indicate higher distress, with 63 or more in the high-risk stress group, 23–62 in the potential stress group, and less than 23 in the healthy group.

#### Connor–Davidson Resilience Scale

Resilience is assessed using the Connor–Davidson Resilience Scale (CD-RISC), which is a 25-item self-report instrument that uses 5-point response scales, as follows: 0 = not true at all, 1 = rarely true, 2 = sometimes true, 3 = often true, and 4 = true nearly all of the time (Connor and Davidson, 2003). The CD-RISC captures how the participant felt over the past month and total scores range from 0 to 100, with higher scores reflecting greater resilience.

#### WHO Quality of Life Scale Abbreviated Version

QOL is measured using the WHO Quality of Life Scale Abbreviated Version (WHOQOL-BREF) (Group, 1998; Min et al., 2002), which defines QOL as an “individual's perception of their position in life in the context of the culture and value systems in which they live and in relation to their goals, expectations, standards, and concerns.” (Skevington et al., 2004; Suh et al., 2015). The WHOQOL-BREF addresses four domains (physical health, psychological health, social relationships, and environmental), as well as general health and overall QOL.

### EEG Recording Features

The participants were seated and engaged in a resting state in an isolated sound-shielded room connected to a recording room *via* a one-way glass window. EEG recordings lasted for 10 min and included the following conditions: 4 min with eyes closed, 2 min with eyes open, and 4 min with eyes closed. All EEG activity was recorded using a 64-channel Quik-cap (Compumedics Neuroscan, El Paso, TX, USA) based on the modified international 10/20 system, in conjunction with vertical and horizontal electrooculograms (EOGs) and one bipolar reference electrode connected to the mastoid. All EEG acquisitions were done using SynAmps 2 (Compumedics, Abbotsford, Australia) and the Neuroscan system (Scan 4.5; Compumedics). EEG signals were amplified at a sampling rate of 1,000 Hz using a 0.1 to 100 Hz online bandpass filter and a 0.1 to 50 Hz offline bandpass filter, while electrode impedance was kept below 5 kΩ.

All acquired EEG data were processed with NeuroGuide software (ver. 2.6.1; Applied Neuroscience, St. Petersburg, FL, USA). For the analyses, 19 of the 64 channels were selected according to the montage set with linked ear references from the NeuroGuide, as follows: FP1, F3, F7, Fz, FP2, F4, F8, T3, C3, Cz, T4, C4, T5, P3, O1, Pz, T6, P4, and O2. All EEG recordings under eyes-closed conditions were selected and artifacts were removed using the artifact rejection toolbox in NeuroGuide based on visual inspection. Artifact removal was performed offline using the artifact rejection toolbox of NeuroGuide software. EEG recordings were also visually inspected to eliminate eye muscle movements and other artifacts, and artifact-free epochs under eyes-closed conditions were selected for spectral analysis. Accepted epochs of EEG data for both absolute (uV^2^) and relative (%) power were smoothed using fast Fourier transforms and averaged in seven frequency bands by NeuroGuide's spectral analysis system: delta (1–4 Hz), theta (4–8 Hz), alpha (8–12 Hz), beta (12–30 Hz), high beta (25–30 Hz), gamma (30–40 Hz), and high gamma (40–50 Hz).

### PET Recording Features

The ^18^F-FDG-PET scans were acquired using a Gemini TF64 PET/CT scanner (Philips Healthcare, Andover, MA, USA). The subjects received an intravenous injection of 4.8 MBq/kg of 18F-FDG in a room with dimmed lights and were instructed to remain to lie comfortably during the FDG equilibration period. The brain emission images were acquired 40 min after the bolus injection of 18F-FDG and continued for 10 min with a 2-mm thickness, 90 slices, and a 256×256 matrix size. Uniform reconstruction protocols were applied to factor out possible sources using the 3D Row-Action Maximum-Likelihood Algorithm in 90 slices with 2 mm thickness in a 128 × 128 matrix. All reconstructed images were corrected for attenuation and scatter.

First, the ^18^F-FDG-PET images of each participant were spatially transformed into the Montreal Neurological Institute (MNI) standard PET template that employs a 12-parameter affine transformation followed by nonlinear deformation. Brain glucose metabolism at each voxel was proportionally scaled to the global mean value to reduce individual variation; hence, the relative regional glucose metabolic rate was calculated. Second, the preprocessed and normalized PET images were parcellated based on the Automated Anatomical Labeling (AAL) template, which divides the brain into 90 anatomical ROIs, except the cerebellum (Tzourio-Mazoyer et al., 2002). Finally, we extracted the mean glucose uptake values from each ROI of the AAL template for all subjects. Preprocessing was performed using Statistical Parametric Mapping (SPM12, Wellcome Department of Imaging Neuroscience, London, UK, http://www.fil.ion.ucl.ac.uk/spm) implemented in MATLAB 9.1 (The MathWorks, Inc., Natick, MA, USA).

### Leave-One-Out Cross-Validation

For the model assessment and prediction evaluation, the best approach is to divide the data set into three parts: training, validation, and test data. However, when the sample size is relatively small, the Leave-one-out cross-validation (LOOCV) approach is often used (Chen et al., 2012; Sun et al., 2014; Zeng et al., 2016). In this study, we also adopt the LOOCV approach to evaluate and report the performance of various methods. Because LOOCV is a special case of K-fold cross-validation, it does not tend to overestimate the test error rate (James et al., 2013).

### Multimodality

**Figure 1:**
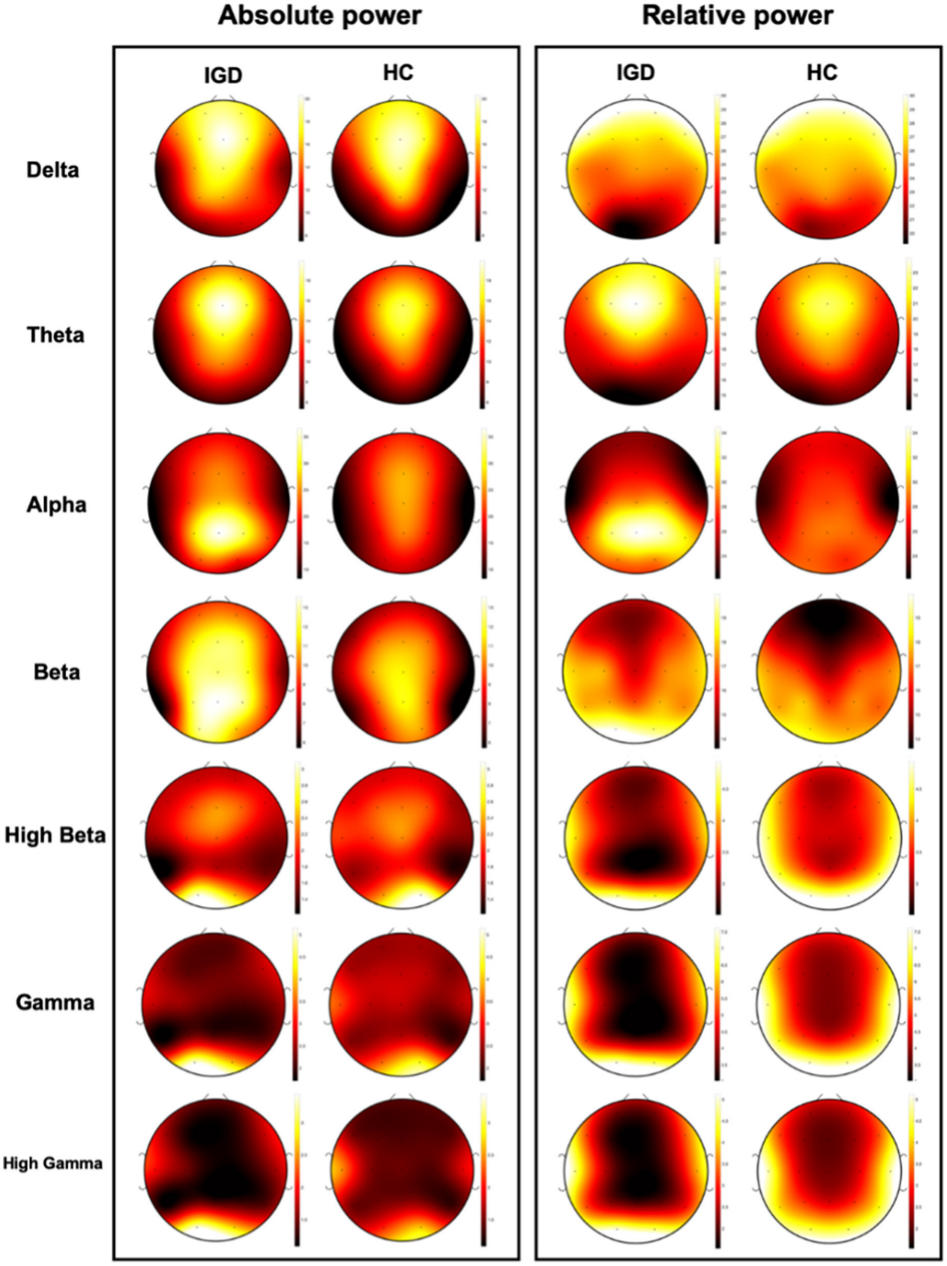
Visualization of mean absolute power and relative power of EEG data for IGD and Health control (HC) group.

**Figure 2:**
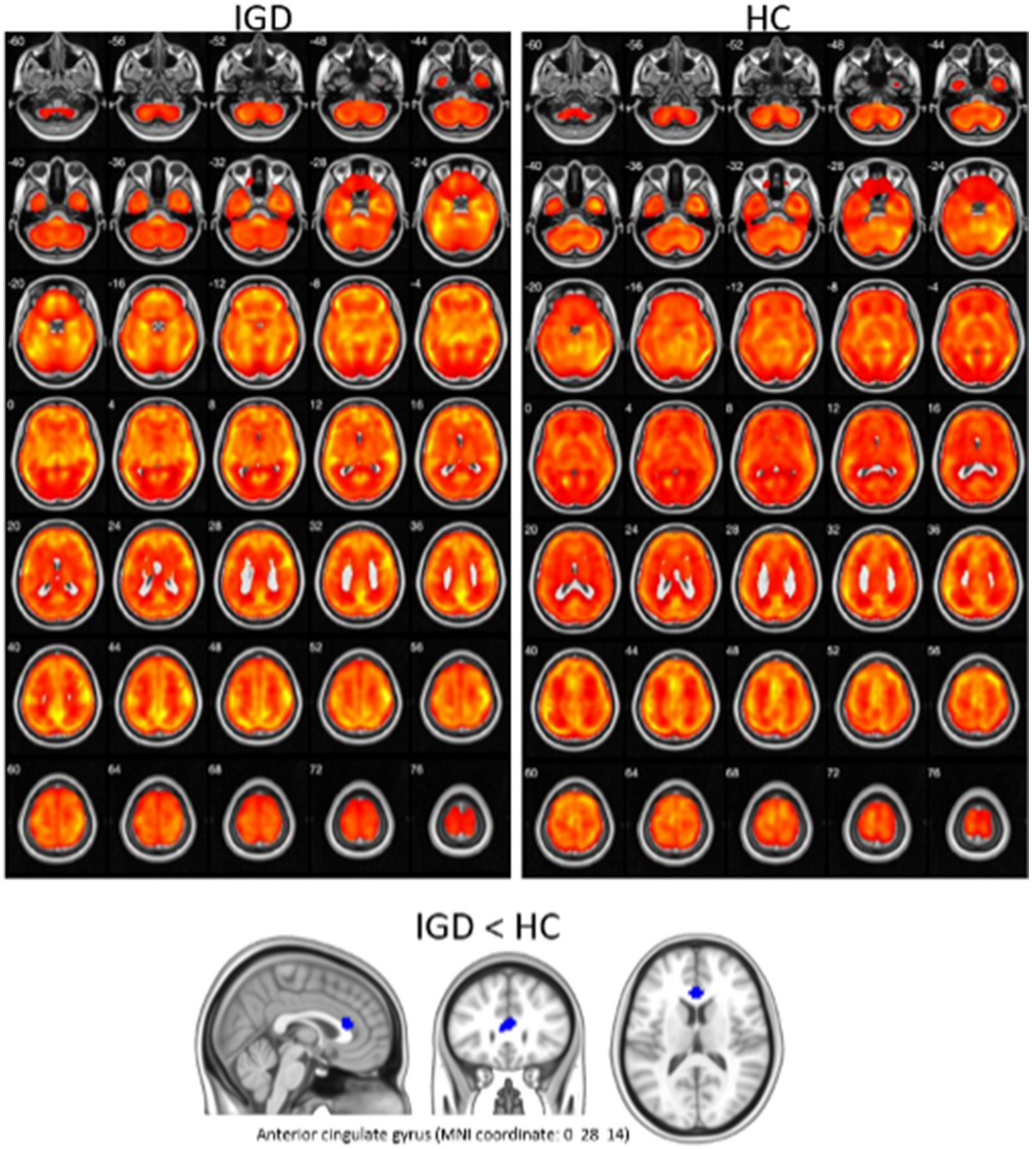
Visualization of mean metabolic uptake of ^18F^-FDG-PET in IGD and HC. Top: areas showing significant glucose metabolism in both IGD and HC, using one-sample t-test (corrected *p* < 0.05, cluster size (*k*) > 100). Bottom: IGD showed lower glucose metabolism in anterior cingulate gyrus, compared with HC (*p* < 0.005 uncorrected, *k* > 100).

**Figure 3:**
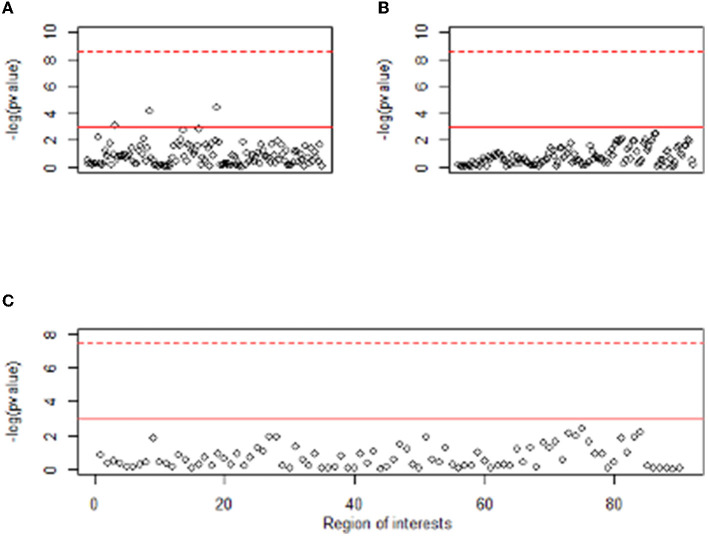
Manhattan plot of t-test result between two groups for EEG and PET features. The y-axis of plots means –log (*p* value). The x-axis of **(A,B)** represents the absolute power and relative power of the EEG, respectively, and the x-axis of **(C)** represents 90 regions of interest of PET. Dashed red line means Bonferroni level of significance and solid red line means 0.05 significance level.

Clinical variables, EEG, and PET are obtained from different sources, and their characteristics are also different. It means that the data have three different modalities, which is called multimodal data. Figure 1 shows the absolute and relative power of EEG for IGD and HC samples and Figure 2 shows the mean metabolic uptake of 18F-FDG-PET in two groups. However, when we conduct a two-sample t-test to compare the mean value of EEG and PET features, there are few significant features statistically (Figure 3). It suggests that EEG and PET modal data may be weak on their own but may play a role in complementing clinical variables. Therefore, it is necessary to properly integrate weak modalities such as EEG and PET with the clinical modal to enhance the performance of the IGD prediction model. The simple way to integrate different modalities is to line up all features into a longer feature vector. However, this does not fully account for the multimodal characteristics of the data and therefore cannot be an efficient integration. More efficient integration of multimodal data can contribute to improving the performance of the prediction model.

### Multiple-Kernel SVM

To reflect the multimodality of the data, we adopted the multiple-kernel SVM (Zhang et al., 2011). It enables efficient integration of multimodal data through the kernel combination, and it is easy to implement because it can be conveniently solved through standard SVM solvers.

At first, the main idea of the standard SVM is to find a linear separating hyperplane that maximizes the margin, that is, the largest distance gap between the two group's data points. For nonlinear separable cases, input data are mapped from their original space to a higher dimensional space through a kernel-induced mapping function by finding a linear hyperplane. The detailed algorithm of standard SVM is as follows.

**Table:** 

Standard Support Vector Machine (SVM)
**Input**
Training set *S* = {(*x*_1_, *y*_1_), …, (*x*_*n*_, *y*_*n*_)}, regularization parameter *C*, specified kernel, and kernel parameters
**Initialization**
Compute the kernel of distances between the datapoints *k*(*x, x*′) = 〈*h*(*x*), *h*(*x*′)〉
**Training**: Maximizing the margin is equivalent to $\underset{\alpha }{\max}\overset{n}{\underset{i=1}{\sum }}\alpha_{i}-\frac{1}{2}\underset{i,j}{\sum }\alpha_{i}\alpha_{j}y_{i}y_{j}k\left({\text{x}}_{\text{i}},{\text{x}}_{\text{j}}\right)$ $\text{subject to }\overset{n}{\underset{i=1}{\sum }}\alpha_{i}y_{i}=0,\;0\leq \alpha_{i}\leq C,\;i=1,\ldots ,n$
**Output**
solution α^*^,
decision function for new test data **z**, represented by hyperplane is
$f\left(z\right)=sign\left(\overset{n}{\underset{i=1}{\sum }}y_{i}\alpha_{i}^{*}k\left(x_{i},z\right)+b\right)$

Based on this standard SVM, multiple-kernel SVM performed the integration of multimodal data by modifying only the kernel function parts while keeping other processes as it is. There, let ${x_{i}}^{\left(m\right)}$ be a feature vector of the *m*^*th*^ modality of the *i*^*th*^ sample. First, calculate each kernel function on the *m*^*th*^ modality, $k^{\left(m\right)}\left({x_{i}}^{\left(m\right)},{x_{j}}^{\left(m\right)}\right)$. Then, combine multiple-kernel matrices into a single kernel matrix which results in mixed kernel $k\left(x_{i},x_{j}\right)=\underset{m}{\sum }\beta_{m}k^{\left(m\right)}\left({x_{i}}^{\left(m\right)},{x_{j}}^{\left(m\right)}\right).\;$In this process, constraint $\underset{m}{\sum }\beta_{m}=1$ is used to make the easy interpretation of modality contributions and grid searches to find β_*m*_s for the optimal prediction model. Finally, using this combined kernel matrix, train a single SVM model and find an optimization solution α^*^, and decision function for classification.

#### Optimal Weights for Kernel

To propose a binary classification rule that predicts whether the subject belongs to the IGD group, an optimal prediction model, that is, the highest prediction performance model should be determined. Finding the optimal prediction model in a multiple-kernel SVM using a combined kernel is the same problem as determining β_*m*_s. To get the optimal β_*m*_s while avoiding overfitting, we adopted the nested cross-validation approach which is frequently used for the small sample case (Dora et al., 2018; Wainer and Cawley, 2021). In the nested cross-validation, we used LOOCV in Section 2.5 as the outer loop and 5-fold cross-validation as the inner loop. In the inner loop, we performed a grid search to find the optimal β_*m*_s in terms of the five-fold cross-validated AUC. After determining the optimal hyperparameter β_*m*_s, we computed the LOOCV AUC of the multiple-kernel SVM for comparing the performance with other methods.

#### Interpretation of Kernel

To find out how the information of each modality is combined to contribute to the predictive performance, it is necessary to focus on the kernel of multiple-kernel SVM. To interpret the combined kernel, first, we visualized three separate kernels from each modality and the combined kernel. In addition, we performed the Principal Component Analysis (PCA) on the kernel matrix to check whether the combined kernel properly contains the information necessary to classify the two groups. Further, we illustrated how the first and second principal components (PCs) obtained from PCA in the kernel matrix classify IGD and HC groups.

### Model Comparison

We conducted various experiments to examine (1) the usefulness of multimodality features compared to considering single modality only and (2) how well the process of multiple-kernel SVM properly integrated different information from multi-modalities. For this purpose, we compared multiple-kernel SVM with conventional machine learning methods such as SVM, Xgboost, and Random Forest with features for each modality or with just stacked features. Recently, many researchers have exploited deep learning in neuroimaging studies since it automatically handles many features in the model. As (Cho et al., 2015) pointed out, small sample size is vulnerable to the high performance of deep learning models, especially in convolutional neural network (CNN), which uses the image as an input. This is because complex deep learning models have a huge number of parameters that must be trained (Brigato and Iocchi, 2021). In general, using a deep learning model is recommended when the sample size is extremely large than other statistical methods (James et al., 2013). Therefore, we focused on machine learning methods, but for comparison, we also considered the multilayer perceptron (MLP) model, a representative model with relatively less complexity.

## Results

### Demographic Statistics

To compare the demographic and clinical characteristics of the IGD and HC groups, an independent two-sample t-test was performed, and the results are shown in Table 1. The mean values of all clinical features except age, BAS, and ECQ were significantly different in the two groups.

**Table 1:** Demographic and clinical characteristics.

	**IGD** **(*****n*** **=** **28)**		**HC** **(*****n*** **=** **24)**		***t***	***p* value**
	**Mean**	**SD**	**Mean**	**SD**		
Age	24.21	5.01	24.25	2.72	0.033	0.974
Y-IAT	63.21	17.00	31.70	9.38	–8.379^*^	<0.001
BDI	16.69	11.16	3.88	4.03	–5.657^*^	<0.001
BAI	13.40	12.24	5.01	6.02	–3.203^*^	0.003
BIS	21.47	3.75	16.97	4.42	–3.924^*^	<0.001
BAS	35.16	7.43	31.90	6.74	–1.660	0.103
AQ	73.43	18.22	56.17	13.82	–3.877^*^	<0.001
BIS-11	66.50	11.12	54.88	7.69	–4.432^*^	<0.001
PWI	64.42	26.90	29.95	15.73	–5.568^*^	<0.001
ECQ	10.64	3.87	10.33	2.77	–0.335	0.739
CD-RISC	49.58	17.32	72.26	9.29	5.996^*^	<0.001
WHOQOL-BREF	48.85	9.35	59.87	7.09	4.824^*^	<0.001

*IGD, internet gaming disorder; HC, healthy controls; SD, standard deviation; Y-IAT, Young's internet addiction test; BIS, Behavioral inhibition system; BDI, Beck depression inventory; BAI, Beck anxiety inventory; BAS, Behavioral Activation system; AQ, Aggression Questionnaire; BIS-11, Barratt impulsiveness scale; PWI, Psychosocial well-being index; ECQ, Emotional Control Questionnaire; CD-RISC, Connor-Davidson resilience scale; WHOQOL-BREF, WHO Quality of Life Scale Abbreviated Version;*

* *p < 0.05*.

### Optimal Multiple-Kernel SVM Model

We need to decide the optimal prediction model to propose a prediction rule in the classification of IGD and HC. The optimal weights for the kernel contribution of PET, clinical variables, and EEG are 0.32 (±0.21), 0.62 (±0.19), and 0.06 (±0.09) respectively. When the optimal kernel weights for multiple-kernel SVM are fixed as average values: 0.32, 0.62, and 0.06, Table 2A shows the classification result based on LOOCV. In this case with the high accuracy of 84.6%, the sensitivity was 89.3%, which was higher than the specificity of 79.2%. To illustrate the effect of the contribution of EEG increases from the optimal case, we choose (0.05, 0.6, and 0.35) where the contribution of EEG is the highest in the nest cross-validation. Table 2B is the classification results with these weights. If the contribution of EEG increases from optimal case 0.06 to 0.35, only two more people are misclassified.

**Table 2:** Proposed prediction rule based on multiple-kernel SVM.

**(A) Optimal case**				**(B) Secondary case**			
***β_*****PET*****_*****=** **0.32**, ***β_*****Clinic*****_*****=** **0.62**, ***β_*****EEG*****_*****=** **0.06**				***β_*****PET*****_*****=** **0.05**, ***β_*****Clinic*****_*****=** **0.6**, ***β_*****EEG*****_*****=** **0.35**			
**Confusion matrix**		**Reference label**		**Confusion matrix**		**Reference label**	
		**HC**	**IGD**			**HC**	**IGD**
Predicted label	HC	19	3	Predicted label	HC	20	6
	IGD	5	25		IGD	4	22
Accuracy: 84.6% Sensitivity: 89.3% Specificity: 79.2%				Accuracy: 80.8% Sensitivity: 78.6% Specificity: 83.3%			

*IGD, internet gaming disorder; HC, healthy controls*.

### Comparison of the Prediction Performance

**Figure 4:**
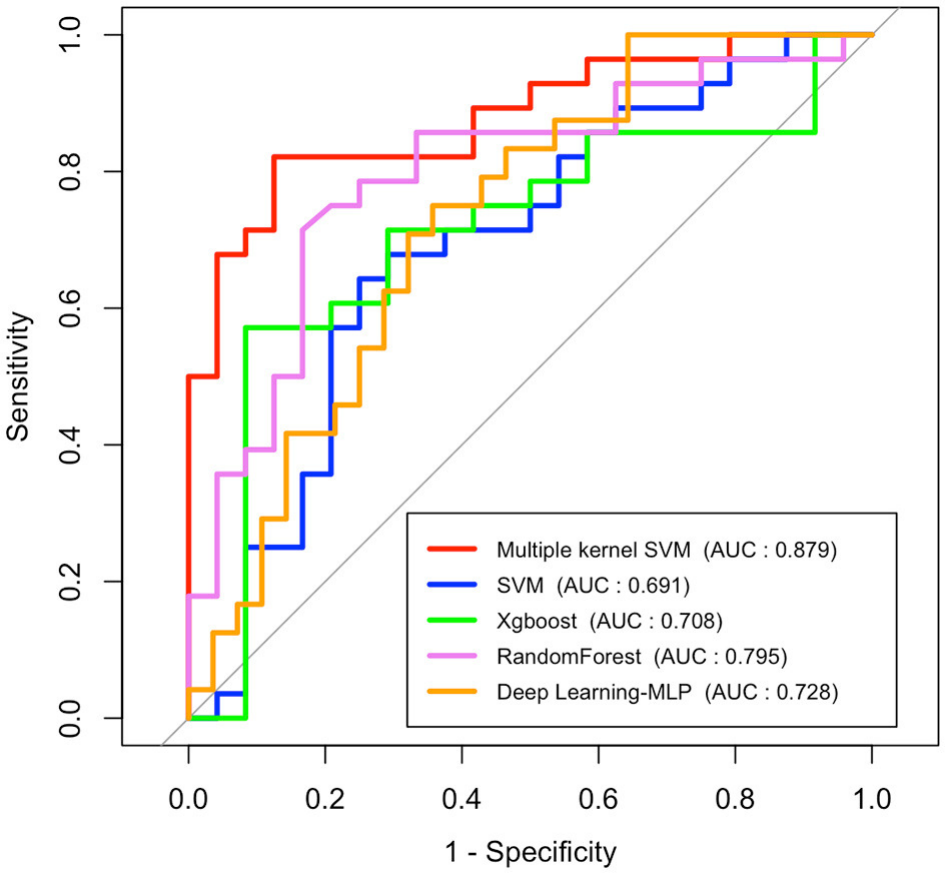
ROC curves and AUC values of conventional machine learning methods, deep learning method (multilayer perceptron model), and multiple-kernel SVM.

To investigate how efficient multimodal data integration using multiple-kernel SVM is, we compared it with other existing machine learning methods such as simple SVM, Xgboost and Random Forest, and deep learning (multilayer perceptron model). These conventional learning methods simply use feature vectors by stacking all modalities. Figure 4 shows the ROC curves of the proposed method and the conventional methods. In terms of the ROC curve and the area under the curve (AUC), the multiple-kernel SVM is the best (AUC =0.884).

**Figure 5:**
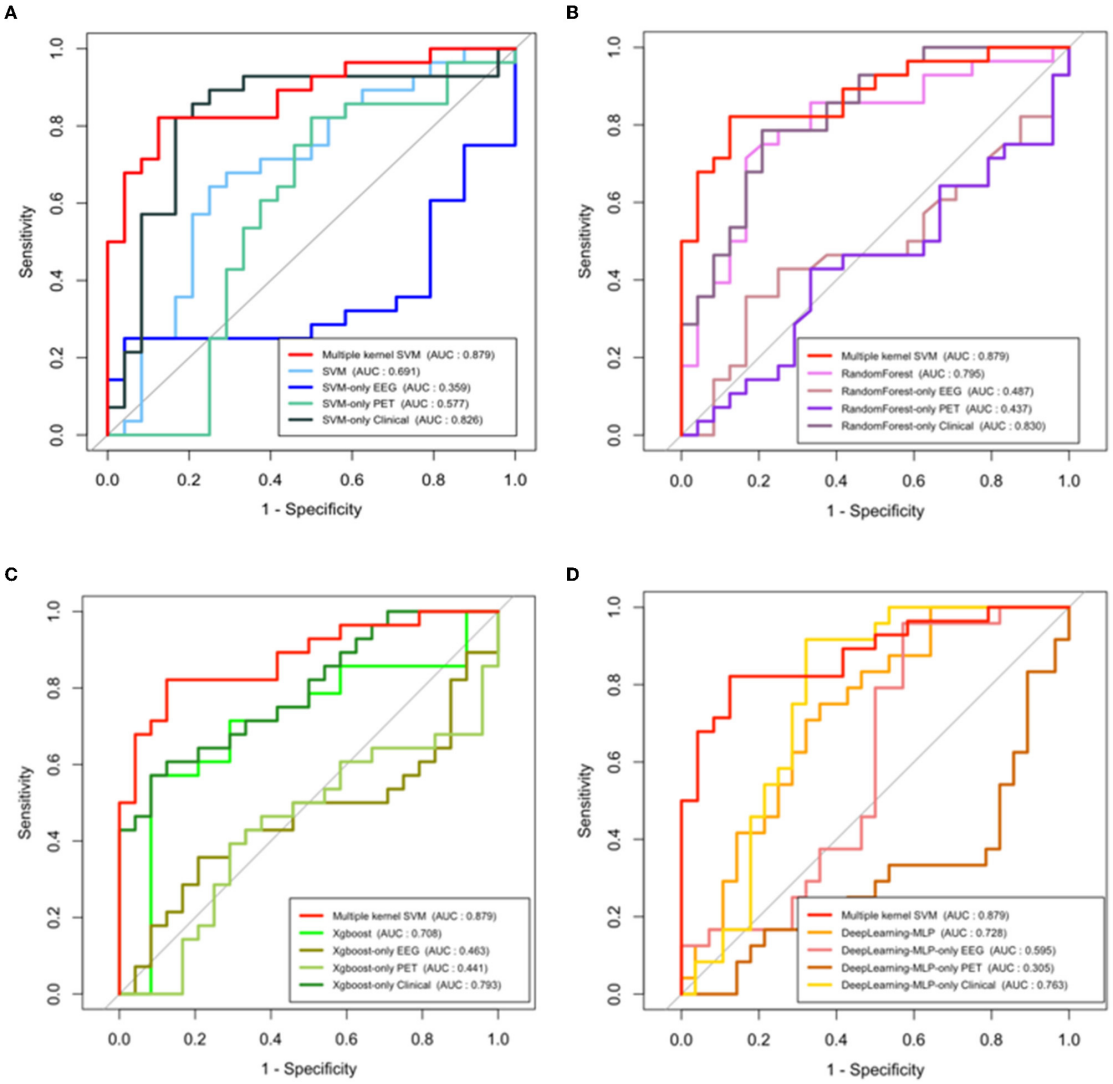
ROC curves and AUC values of single modal models and multiple-kernel SVM with each conventional machine learning methods. **(A)** SVM, **(B)** random forest, **(C)** Xgboost, and **(D)** deep learning (multilayer perceptron model).

Also, to highlight the superiority of the use of multimodal data, we examined the performance of the single-modality model. Figure 5 represents the ROC curves of each modality which uses only a single type of feature as an input in SVM (a), Random Forest (b), Xgboost (c), and deep learning (d), respectively. In addition, we compared the performance of the model which uses all features as input in a single line, and the proposed multiple-kernel SVMs. When either EEG or PET are used, the AUCs of all single-modality models are low (around 0.5). Although the single-modality model with clinical features only shows relatively higher accuracy, the multiple-kernel SVM outperforms all single-modality methods considered in this study.

### Interpretation of Combined Kernel Matrix

**Figure 6:**
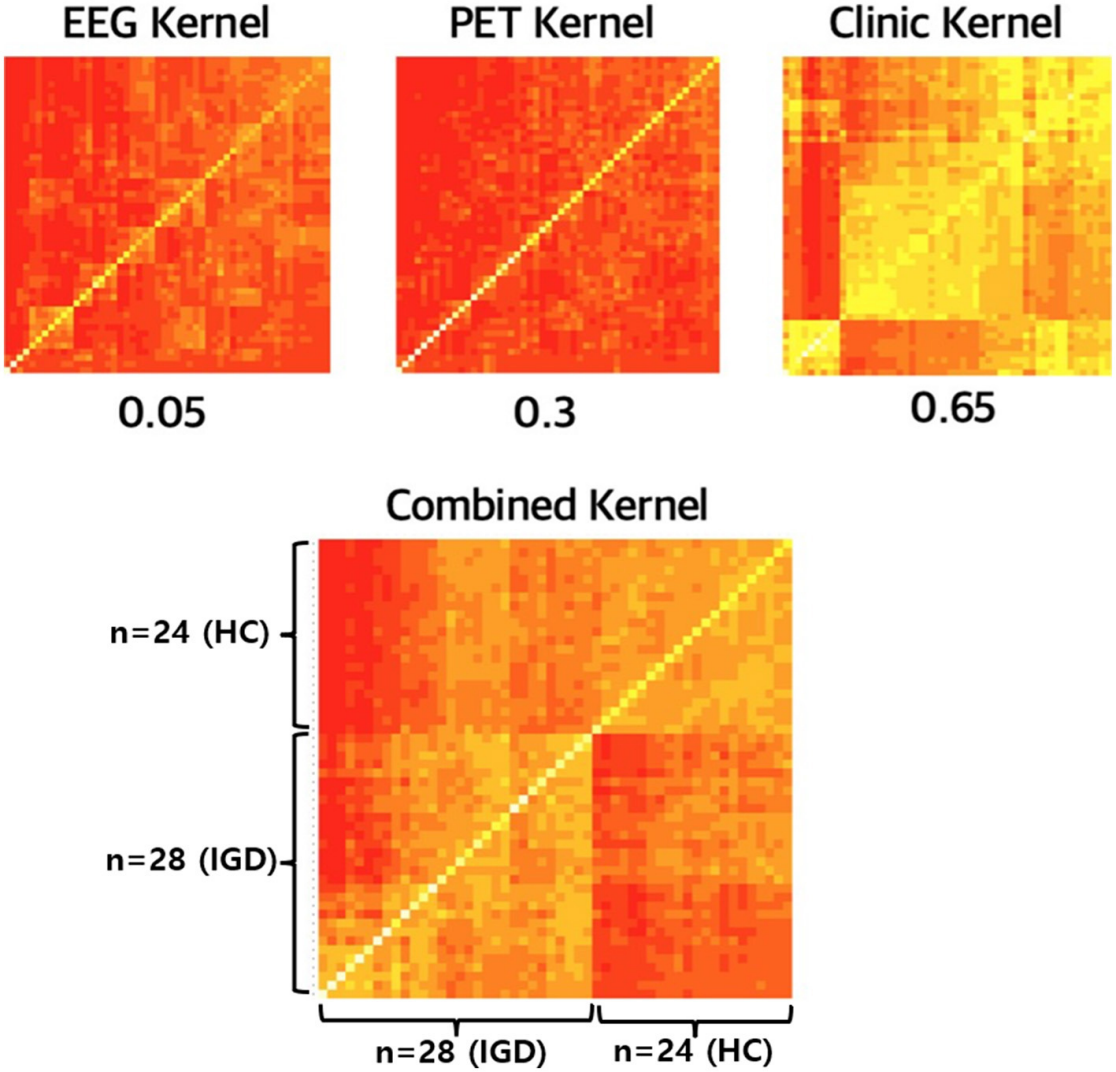
The kernel matrices for EEG, PET, and clinical features and its combined kernel.

Up to now, we focus on the prediction performance of multiple-kernel SVM. In the fitting process, the kernels for each modality are combined and it plays a key role in multimodal data integration. Figure 6 shows the kernel matrices for three modalities, EEG, PET, and Clinical features, and the combined kernel matrix of the optimal multiple-kernel SVM. Kernel matrix represents the distance, that is, the similarity between two samples. That is, in Figure 6, the lower-left part of the matrix represents the similarity between IGD groups, and the upper-right part of the matrix represents that of HC groups. In contrast, the upper-left and lower-right parts of the matrix represent the similarity between different groups, IGD-HC. Therefore, if the kernel matrix properly works for two separate groups, the entire kernel matrix should show four distinct parts. Each kernel made from only EEG or PET does not have clear separation, respectively. Also, the kernel made from clinical variables does not have proper separation. However, in the combined kernel matrix with optimal weights, IGD and HC groups are properly well-separated. In Figure 6, when the similarity is low, the color of the corresponding element is closer to red. When we compare the lower-left part (IGD-IGD) and the lower-right part (IGD-HC), the lower-right part, which shows the similarity between the different groups, is more reddish. Therefore, the kernel matrix correctly expresses the distance between heterogeneous groups.

**Figure 7:**
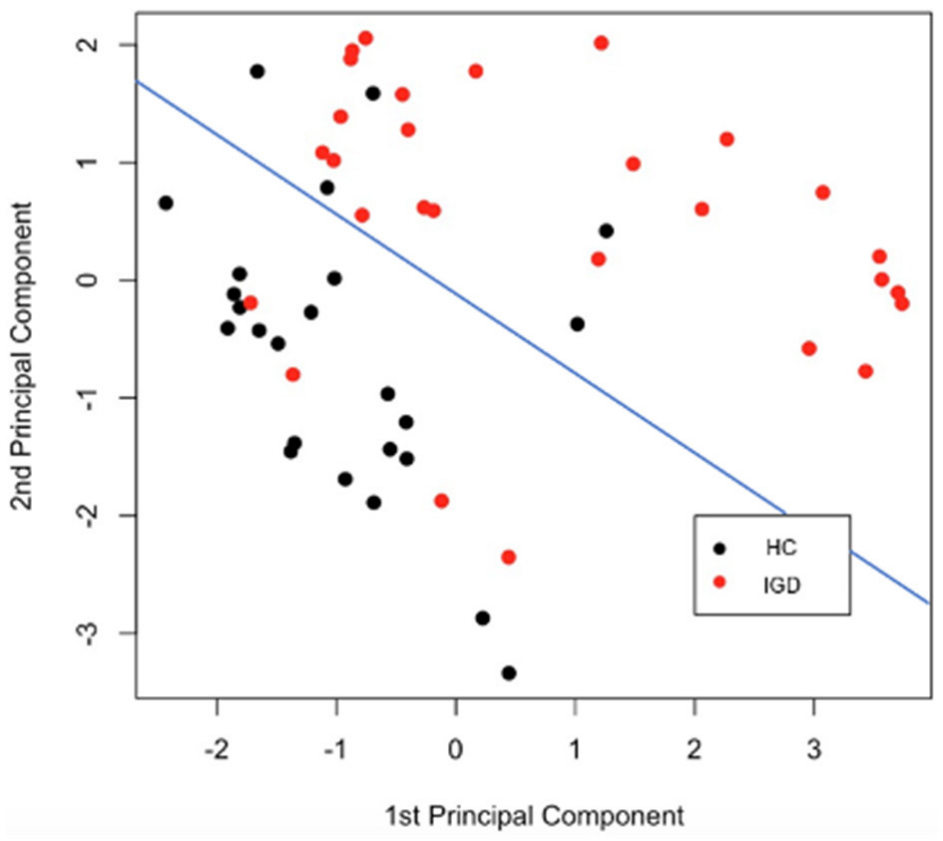
Kernel PCA using combined kernel. HC, healthy control; IGD, internet gaming disorder.

Figure 7 is a diagram showing how data belonging to each group is represented when the two principal components obtained from the kernel PCA result are taken as axes. After the kernel-mapped high-dimensional features are reduced to two PC components, the pairs of the first and second PCs are well-separated into IGD and HC groups. The blue line drawn in Figure 7 shows the separation plane obtained after fitting the logistic model that classifies the two groups using only the two principal components as explanatory variables.

## Discussion

The present study proposed an IGD prediction model by integrating each distinct modality including clinical feature, EEG, and PET to enhance prediction accuracy. We adopted a multiple-kernel SVM that reflected multimodality by combining the calculated kernels from each modality to create one mixed kernel. Compared to the conventional methods, our proposed prediction rule achieved more than 80% accuracy, sensitivity, and specificity, which can be applied to the real world. This suggests that proper integration of multimodal data contributed to the construction of the prediction model for IGD.

We noted that the conventional Xgboost, Random Forest, SVM, and MLP used a single input vector by stacking all features. The prediction performance from popular machine learning and deep learning techniques is significantly lower than that of multiple-kernel SVM, which means that the method of combining the features into a long feature vector is not a way of fully integrating data information. Furthermore, the fact that the performance of a model generated using only clinical features is higher than that of a model using all features in batches means that processing multimodal data using such a long vector impairs the performance of the model. Contrary to this, if an appropriate integration process is used, weak modalities such as EEG and PET, which cannot individually create meaningful predictive models, can contribute to the improvement of model performance. When predicting IGD, it is important to take advantage of the characteristics of each clinical feature, EEG, and PET modality and incorporate them into sufficient information to complement each other. Although we know clearly that the features come from different sources, in the process of creating one long feature vector, the information about the modal to which each feature belongs is diluted. On the other hand, the multiple-kernel SVM creates a kernel that uniquely reflects the characteristics of each modal to compensate for the distance and characteristics shared by the features of specific sources. In addition, these kernels complement each other in the process of combining them at the optimal ratio for given multimodal data.

It is also shown in the kernel matrix that each weak modality can complement each other and data integration is important for identifying IGD. Multiple-kernel SVM changes only the kernel function of the existing SVM process, so the core part that reflects the multimodality is the combined kernel. The kernel matrix contains information about the distance between the data points. Therefore, when visualizing the kernel matrix, it is recommended that the entire data points are divided into two groups for proper IGD identification. As Figure 6 shows, the kernels made from each modality are not properly divided into two groups. This means that each kernel does not contribute well to the IGD identification. However, in the case of combined kernels, the near and far distances between subjects are distinguished, which means that the combined kernel relatively well contains the information which is necessary to classify the two groups.

Another advantage of integrating multimodal data by combining the kernels is that it is interpretable. Once we find the optimal model, we can find kernel weights, which in turn indicate how much each modality contributes. In the optimal prediction model, the contribution to the combined kernel is highest at the clinic feature modality (0.65). Relatively, the contribution of EEG and PET kernel was lower than that of clinic features. This is similar to the t-test results where many clinical features have a relatively strong signal, and the EEG and PET feature appears to have weak information in IGD and HC group identification. EEG provides us with important clinical implications, including objective responses and higher temporal resolution images in the brain compared with self-report, as well as being less intrusive for subjects and more cost-effective than PET. Although the EEG features are high-dimensional, the optimal weight for the EEG kernel is relatively lower than the others. After multiple corrections, there are no significant features among EEG. Furthermore, the AUC of the prediction model using only EEG was between 0.3 and 0.6. Thus, it seems that the low kernel weight of EEG stems from the ratio of significant information among the total EEG features is not large. Nevertheless, the contribution of the EEG and PET kernel is not zero, respectively, suggesting that these are indispensable. In the clinical session, the clinical features based on the self-administrated report are frequently used to diagnose IGD. However, we found that the integration with objective methodologies including EEG and PET is necessary for predicting IGD with higher accuracy.

This study is limited by the relatively small number of subjects used for modeling. For integrative analysis, only people with all three clinical, EEG, and PET data collected can be used for the analysis. Fifty-two people collected all three data sets, and taking this into account, the model was evaluated based on LOOCV. But if more samples are available, classification rules can be proposed by making more robust and reliable prediction models. Another limitation is that it is difficult to find a specific feature that has a high contribution to the prediction. Since the existing SVMs focus on increasing classification performance itself, there are few tools to extract features that contribute most to prediction. Therefore, although the contribution of the kernel is known, it is difficult to know the extent to which one specific feature contributes to the prediction model. However, we know that the present study is the first study attempting to predict IGD using multiple-Kernel SVM for integrating several methods and comparing it with other conventional machine learning methods.

In summary, the present study suggests that integrating each method including self-administrated reports, EEG, and PET is useful in predicting IGD. This study is a novel attempt to integrate each method from different sources and suggest a new optimal prediction model for IGD, which helps clinicians to give a precious diagnosis to patients with IGD. Future studies are necessary to assess the value of different data combinations, including neurocognition, connectivity features measured by MRI, PET, or EEG, and multi-omics information in the field of addictive disorders.

## Data Availability Statement

The raw data supporting the conclusions of this article will be made available by the authors, without undue reservation.

## Ethics Statement

The studies involving human participants were reviewed and approved by the Institutional Review Board of SMG-SNU Boramae Medical Center, Seoul, and Republic of Korea. The patients/participants provided their written informed consent to participate in this study.

## Author Contributions

BJ, JL, DL, and J-SC contributed to analyzing data, interpretation of the findings, and writing the manuscript. JL contributed to data collection. BJ, SG, and DL contributed to data analysis. HK, YK, SY, DL, and J-SC contributed to study conception and design, interpretation of the findings, and supervision. All authors critically reviewed content and approved the final manuscript.

## Funding

This work was supported by a grant from the National Research Foundation of Korea (Grant Nos. 2021R1F1A1046081 to J-SC and 2016R1A6A3A11931862 to HK). DL was partially supported by the National Research Foundation of Korea (NRF) grant funded by the Korean government (MSIT) (No. 2021R1A2C1012865).

## Conflict of Interest

The authors declare that the research was conducted in the absence of any commercial or financial relationships that could be construed as a potential conflict of interest.

## Publisher's Note

All claims expressed in this article are solely those of the authors and do not necessarily represent those of their affiliated organizations, or those of the publisher, the editors and the reviewers. Any product that may be evaluated in this article, or claim that may be made by its manufacturer, is not guaranteed or endorsed by the publisher.
